# Evaluation of ICON Maxx, a long-lasting treatment kit for mosquito nets: experimental hut trials against anopheline mosquitoes in Tanzania

**DOI:** 10.1186/s12936-015-0742-z

**Published:** 2015-05-30

**Authors:** Patrick K. Tungu, Robert Malima, Frank W. Mosha, Issa Lyimo, Caroline Maxwell, Harparkash Kaur, William N. Kisinza, Stephen M. Magesa, Matthew J. Kirby, Mark Rowland

**Affiliations:** National Institute for Medical Research, Amani Medical Research Centre, Muheza, Tanzania; Pan-African Malaria Vector Research Consortium (PAMVERC), P.O.Box 81, Muheza, Tanga, Tanzania; Kilimanjaro Christian Medical Centre, Moshi, Tanzania; London School of Hygiene and Tropical Medicine, London, UK

**Keywords:** Long-lasting insecticidal nets, Lambda-cyhalothrin, *Anopheles gambiae*, *Anopheles funestus*, *Anopheles arabiensis*, Experimental huts

## Abstract

**Background:**

Insecticide-treated nets are the primary method of preventing malaria. To remain effective, the pyrethroid insecticide must withstand multiple washes over the lifetime of the net. ICON® Maxx is a ‘dip-it-yourself’ kit for long-lasting treatment of polyester nets. The twin-sachet kit contains a slow-release capsule suspension of lambda-cyhalothrin plus binding agent. To determine whether ICON Maxx meets the standards required by the World Health Organization Pesticide Evaluation Scheme (WHOPES), the efficacy and wash fastness of ICON Maxx was evaluated against wild, free-flying anopheline mosquitoes.

**Methods:**

ICON Maxx was subjected to bioassay evaluation and experimental hut trial against pyrethroid-susceptible *Anopheles gambiae*, *Anopheles arabiensis* and *Anopheles funestus*. Mosquito mortality, blood feeding inhibition and personal protection were compared between untreated nets, conventional lambda-cyhalothrin treated nets (CTN) washed either four times (cut-off threshold) or 20 times, and ICON Maxx-treated nets either unwashed or washed 20 times.

**Results:**

In bioassay, ICON Maxx demonstrated superior wash resistance to the CTN. In the experimental hut trial, ICON Maxx killed 75 % of *An. funestus*, 71 % of *An. gambiae* and 47 % of *An. arabiensis* when unwashed and 58, 66 and 42 %, respectively, when 20 times washed. The CTN killed 52 % of *An. funestus*, 33 % of *An. gambiae* and 30 % of *An. arabiensis* when washed to the cut-off threshold of four washes and 40, 40 and 36 %, respectively, when 20 times washed. Percentage mortality with ICON Maxx 20 times washed was similar (*An. funestus*) or significantly higher (*An. gambiae*, *An. arabiensis*) than with CTN washed to the WHOPES cut-off threshold. Blood-feeding inhibition with ICON Maxx 20 times washed was similar to the CTN washed to cut-off for all three species. Personal protection was significantly higher with ICON Maxx 20 times washed (66-79 %) than with CTN washed to cut-off (48-60 %).

**Conclusions:**

Nets treated with ICON Maxx and washed 20 times met the approval criteria set by WHOPES for Phase II trials in terms of mortality and blood-feeding inhibition. This finding raises the prospect of conventional polyester nets and other materials being made long-lastingly insecticidal through simple dipping in community or home, and thus represents a major advance over conventional pyrethroid treatments.

## Background

Insecticide-treated nets (ITNs) are the most effective and feasible means of preventing malaria in Africa south of the Sahara [1]. Because conventional ITNs need to be re-treated with pyrethroid insecticide at least once per year to maintain their efficacy, several manufacturers of nets have developed long-lasting insecticidal nets (LLINs) in which wash-resistant formulation of insecticide is coated or incorporated into the netting fibres during production [2]. With good LLIN technology, insecticidal efficacy can be maintained against anopheline mosquitoes for at least three years without need for further re-treatment [2]. The advent of LLINs provided a technical solution to the problem of low annual re-treatment rates of conventional ITNs after initial distribution and washing [3] and henceforth LLINs would become the most important tool for malaria prevention in Africa and Asia.

In 2005 the World Health Assembly (WHA) set a target of 85 % of those at risk of malaria should benefit from preventive interventions by the end of 2015 [4]. This led to increased demand for LLINs by national malaria control programmes (NMCPs) to meet the target of at least 85 % protected by 2015, and led to international donors opting for LLIN as their preferred choice of net [5, 6]. The proportion of the population with access to ITNs has increased markedly in sub-Saharan Africa over the ten years since the WHA set the agenda. Based on data from household surveys and reports on ITNs delivered by manufacturers and distributed by NMCPs, an estimated 49 % of the population at risk had access to an ITN in their household in 2013, compared to 3 % in 2004 [7]. Despite this achievement, not all households have enough nets to meet family needs: an estimated 71 % of households have insufficient ITNs to protect all household members and one-third of households do not own even a single ITN [7]. More needs to be done to reach all families with ITNs, and supply enough ITNs for all household members [7].

While the main emphasis has been to treat LLINs during manufacture, the majority of ITNs available through the commercial retail sector are not LLINs and those which are in use from this source have either never been treated or were treated only on purchase [2, 8, 9]. Many households still use locally sourced nets which are not LLINs and which require regular re-treatment with insecticide, when insecticide becomes depleted after repeated washing. Thus, there is a need for a long-lasting insecticide treatment kit which could convert untreated nets into ITNs that can withstand repeated washing without the need for re-treatment. Such an insecticide kit could also be bundled with untreated nets on purchase and enable local producers that lack LLIN manufacturing technology to produce an ITN which could contribute usefully to malaria control and address local LLIN shortages [2, 10].

Two brands of long-lasting treatment kit have so far been developed: KO-Tab 1-2-3 developed by Bayer Environmental Sciences [10] and ICON Maxx, developed by Syngenta [11]. ICON Maxx is based on the slow-release capsule suspension (CS) formulation of lambda-cyhalothrin that has previously been evaluated by WHOPES and recommended for treatment of mosquito nets [12]. ICON Maxx is presented as a twin-sachet pack, containing lambda-cyhalothrin 10CS and binding agent, sufficient for the treatment of an individual polyester mosquito net. The target dose depends on the net size and can range from 50 mg AI/m^2^ for a large family-size net to 83 mg AI/m^2^ for a single-size net. A safety assessment of ICON Maxx concluded that no unacceptable exposures were found in the preparation, maintenance and use of the nets [12].

To determine whether ICON Maxx treated nets meet the standards required by WHOPES, the efficacy and wash fastness of ICON Maxx was evaluated in laboratory and field conditions against wild, free-flying anopheline mosquitoes. This paper reports upon the Phase II experimental hut evaluations undertaken in Tanzania by the National Institute for Medical Research (NIMR) in Muheza against *Anopheles gambiae* and *Anopheles funestus* and by the Kilimanjaro Christian Medical College (KCMCo) in Moshi against *Anopheles arabiensis*. Together, these trials contributed to the WHOPES recommendation for use of ICON Maxx as a long-lasting, wash-resistant treatment for polyester mosquito nets.

## Methods

### Study areas and experimental huts

The study made use of experimental hut sites in two districts of Tanzania: Muheza in Tanga region and Moshi in Kilimanjaro region. The Muheza trial was conducted at the NIMR field station at Zeneti village 5° 13’ S latitude, 38° 39’ E longitude and 193 m altitude; where *An. gambiae* s.s. and *An. funestus* are the major malaria vectors [13]. Insecticide susceptibility tests carried out by NIMR showed that the vector populations were 95-100 % susceptible to alphacyano pyrethroids [14]. The Moshi trial was conducted at the field site of KCMCo in an area of rice irrigation 3° 23’ S latitude, 37° 20’ E longitude and 800 m altitude; where *An. arabiensis* is the vector species. Insecticide susceptibility tests indicated susceptibility to alphacyano pyrethroids [15].

The huts at both sites were constructed to a design described by World Health Organization (WHO) [16] based on the original verandah-hut design developed in Tanzania [17, 18]. Modifications included a reduced eave gap of 2 cm, a wooden ceiling, a roof of corrugated iron, and a concrete floor surrounded by a water-filled moat. The huts had open eaves with verandah traps and window traps on each side. The working principle of these huts has been described previously [19].

### Net preparation and washing

ICON Maxx is a twin-sachet kit, with one containing 7.3 ml of lambda-cyhalothrin 10 % CS and the other containing 7.7 ml of binding agent. The target dose of lambda-cyhalothrin on a family size (130 × 180 × 150 cm) polyester mosquito net is 55 mg AI/m^2^ (corresponding to 1.55 g AI/kg for a 100-denier net). The ICON Maxx kits and a white coloured 100-denier family-size nets used in the study were supplied by Syngenta (Basel, Switzerland). During treatment, the contents of both sachets were mixed with 500 ml of water, sufficient to saturate an individual polyester family-size net.

Conventionally treated family-size nets were treated with lambda-cyhalothrin 2.5 % CS (Iconet®, Syngenta; Basel, Switzerland) to a target dose of 15 mg/m^2^ recommended by WHO [20]. To simulate wear and tear a total of six 4 cm × 4 cm holes were cut into each net (two holes on each side and one hole at each end). The long-lasting insecticidal nets (LN) and conventional lambda-cyhalothrin treated nets (CTN) were washed according to WHOPES Phase II washing protocols [16]. Each net was washed individually in 10 l of tap water containing 2 g/l of soap (‘Savon de Marseille’), subjected to 20 rotations per min for 6 min during a 10 min immersion, then rinsed twice. The interval between washes was one day, which is the established regeneration time for ICON Maxx [12]. The washing schedule was stepped to ensure that the final wash of all treatment arms of the trial was completed on the same day.

The CTN washed to the ‘point of insecticide exhaustion’ served as a positive control against which to assess ICON Maxx performance. The point of insecticide exhaustion or cut-off point, as defined by WHOPES, is the number of washes at which the net causes less than 80 % mortality and 95 % knock-down in WHO cone bioassays conducted after each wash [16]. Determination of the point of exhaustion was carried out by exposing unfed *An. gambiae s.s*. Kisumu in ten replicates of five mosquitoes after each wash interval on the five panels of the CTN. Exposure was for 3 min, knock-down was scored after 60 min and mortality was scored 24 hr later. The same procedure was adopted for a ICON Maxx treated net to determine the number of washes which ICON Maxx treatment causes less than 80 % mortality and 95 % knock-down in WHO cone bioassays conducted after each wash.

### Experimental hut study design

The following five treatment arms were tested in the huts: (i) unwashed ICON Maxx net, (ii) ICON Maxx net washed 20 times, (iii) polyester net conventionally treated with lambda-cyhalothrin at 15 mg/m^2^ and washed four times, (iv) polyester net conventionally treated with lambda-cyhalothrin at 15 mg/m^2^ and washed 20 times, (v) untreated unwashed polyester net.

The primary outcomes were: (i) deterrence - the reduction in entry into treatment hut relative to the control huts (i.e., those containing untreated nets); (ii) treatment induced exiting - the proportion of mosquitoes found in exit traps of treatment huts relative to the same proportion in control huts; (iii) mortality - the proportion of mosquitoes killed relative to the total catch size; (iv) overall killing effect - the numbers killed by a treatment relative to the untreated control, as derived from the formula: *Killing effect* (*%*) *= 100* (*Kt*-*Ku*)*/Tu*, where; (i) Kt is the number killed in the huts with treated nets, (ii) Ku is the number dead in the huts with untreated nets, and (iii) Tu is the total entering the huts with untreated nets; (v) blood-feeding inhibition - the proportional reduction in blood feeding in huts with treated nets relative to controls with untreated nets; and (vi) personal protection - the reduction in mosquito biting by treated nets relative to untreated nets, as derived from the formula: *% Personal protection = 100* (*Bu-Bt*)*/Bu*, where (i) Bu is the total number blood-fed mosquitoes in the huts with untreated nets, and (ii) Bt is the total number blood-fed in the huts with treated nets.

Each morning dead and live mosquitoes were collected from the verandahs, rooms and window traps. Live mosquitoes were provided with 10 % sugar solution. Delayed mortality was recorded after 24 hours. Mosquitoes were identified to species and gonotrophic status was recorded as unfed, blood-fed, semi-gravid, or gravid.

Experimental hut trials were conducted in Muheza and Moshi to similar study design. Latin squares were adopted to adjust for any variation between hut position, volunteer sleeper attractiveness and individual nets. The treatment arms were rotated once through each of the huts: a treatment was assigned at random to a particular hut for six (Muheza) or four (Moshi) nights of observation before being transferred to the next hut. Between 19:30 and 06:30 hours adult volunteers slept on beds under the nets. The sleepers were rotated through the huts on consecutive nights. Two to three nets were available per treatment arm and each net was tested for two nights during the four- or six-night rotation. At the end of the rotation the huts were cleaned and aired for one day before starting the next rotation. Data were collected for 36 nights in the Muheza trial and for 24 nights in the Moshi trial.

Random samples of *An. gambiae s.l*. from the huts were identified to species by polymerase chain reaction (PCR) [21]. Species identification recorded 100 % *An. gambiae s.s*. from Zenet, Muheza (N = 60) and 100 % *An. arabiensis* from Lower Moshi (N = 60). Based on these results all specimens collected in the hut trials were recorded as *An. arabiensis* in Moshi and as *An. gambiae s.s*. in Muheza.

The criterion for efficacy was that the ICON Maxx washed 20 times should perform equal to or approximate number of washes a LLIN is likely to incur during its lifetime.

### Chemical analysis

The lambda-cyhalothrin content of ICON Maxx and CTN nets used in the hut trials was estimated from netting samples (four per net) cut before and after washing according to WHO guidelines [15]. Lambda-cyhalothrin was extracted using acetonitrile and injected onto high performance liquid chromatography (HPLC) (Dionex Summit, Surrey, United Kingdom), separated on a 120 Å column, eluted with a 10 % acetonitrile aqueous solution and passed through a PDA-100 detector at 275 nm. From the calibration curve, the lambda-cyhalothrin content and the dosage per m^2^ was calculated.

### Supporting bioassay tests on ICON Maxx nets and CTNs used in the trials

#### Cone bioassays

Efficacy of ICON Maxx and CTN was assessed using WHO cone bioassays after treatment, after completing the washing cycles and at the end of the hut trials. Bioassay tests were conducted using a total of 50 *An. gambiae* Kisumu (pyrethroid susceptible), two to five days of age, on five sections of the net as per WHO guidelines in conditions of 25 ± 2 °C and 75 ± 10 % humidity. Mortality was recorded 24 hours after exposure.

#### Tunnel tests

The tunnel tests were carried out on pieces of ICON Maxx and CTN netting taking from the hut trials nets after 0, 20 and 30 washes. The additional washing to 30 washes was to determine whether the long-lasting treatment could withstand more than the standard 20 washes. The tests were conducted at the KCMCo Moshi site using laboratory-reared *An. arabiensis* Doldotha strain (pyrethroid susceptible).

The standard WHO tunnel test was modified by inserting a transverse paper screen, with a 10 cm diameter hole, across the mosquito release chamber between the point of release and netting insert. The purpose was to prevent mosquitoes from contacting the net except after undertaking host orientation flights. Otherwise the tunnel test apparatus was standard, being comprised of a glass cylinder, 25 cm high, 21 cm wide, 60 cm long, divided into two by a transverse insert made of test netting. Nine 1 cm diameter holes were cut into the netting to allow passage of mosquitoes to the bait chamber. In the bait chamber, a guinea pig was housed unconstrained in a cage and in the release chamber 100 unfed female mosquitoes aged five to eight days were released at dusk and left overnight in conditions of 25 ± 2 °C and 80 ± 10 % humidity. The following morning the numbers of mosquitoes found live or dead, fed or unfed in each compartment were scored and delayed mortality recorded after a further 24 hours [16].

#### Ethics, consent and permission

Ethical clearance was obtained from the ethics committees of the NIMR Tanzania (Ref: NIMR/HQ/R.8a/Vol X/86) and London School of Hygiene and Tropical Medicine (LSHTM). Written informed consent was obtained from all volunteers participating in the study and each was provided with chemoprophylaxis and monitored daily for fever or possible adverse events due to insecticide exposure from the nets.

#### Statistical analysis

The main outcomes were the comparisons of efficacy of the ICON Maxx unwashed and 20 times washed relative to the CTN washed to cut-off in terms of the proportions of mosquitoes blood-feeding or killed by the treatments. Logistic regression analysis was used to estimate proportional outcomes (mortality, blood-feeding, exiting) and negative binomial regression was used to analyse counts of mosquitoes blood feeding (personal protection) or dying (overall insecticidal effects) relative to the untreated control, after adjusting for variation between individual sleepers and hut position. Laboratory bioassay data was analysed using logistic regression.

## Results

### Determination of the cut-off number of washes for conventional treated net

**Fig. 1:**
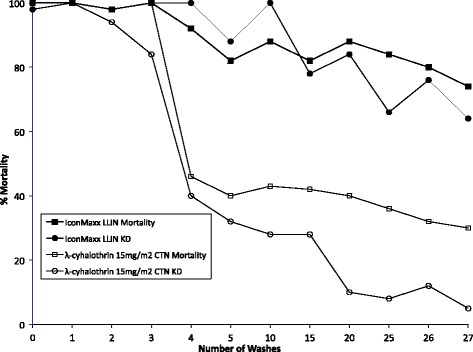
Mortality of *Anopheles gambiae* Kisumu exposed in three-min cone bioassays to ICON Maxx LN and lamda-cyhalothrin CTN at 15 mg/m^2^

The cut-off point, sometimes known as the ‘point of insecticide exhaustion’, is the number of washes at which cone bioassay mortality using *An. gambiae* Kisumu still causes ≥80 % mortality [16]. At four washes, mortality fell below the critical threshold with the CTN (Fig. 1) meaning that lambda-cyhalothrin CTN washed three times was the standard reference. With the ICON Maxx treated net the mortality did not fall below the critical thresholds until 26 washes.

### Phase II - experimental hut trials

#### Mosquito entry and exiting from experimental huts

The numbers and proportion entering and exiting the hut are shown in Table 1. During the trial in Muheza, 97 *An. gambiae* and 222 *An. funestus* were collected in the control huts. Percentage deterrence of *An. gambiae* was similar with treatments ICON Maxx unwashed or ICON Maxx 20 times washed (58 *vs* 61 %) and these were not significantly different to CTN washed to the cut-off point (41 %). Deterrence was lowest with the CTN washed 20 times (12 %). With *An. funestus*, the deterrence effect was significantly higher with ICON Maxx 20 times washed compared with the CTN washed to cut-off (66 and 25 %, respectively, P = 0.001). Deterrence was negligible with the CTN washed 20 times (1.8 %). During the trial in Moshi, 483 *An. arabiensis* were collected in the control huts. No significant deterrent effect was observed for any treatment arm.

**Table 1:** Anopheline mosquitoes collected and exiting into verandah and window traps in the ICON Maxx experimental hut trials in Muheza and Moshi, Tanzania in 2008

	Untreated net	ICON Maxx	ICON Maxx	CTN	CTN
Number of washes	0	0	20	Cut-off	20
***Anopheles funestus***					
Total females caught	222	122	76	167	218
Average catch per night	6.2^a^	3.4^b^	2.1^b^	4.6^a^	6.1^a^
% deterrence	-	45	65.8	24.8	1.8
Total females exiting	200	106	71	164	200
% exiting	90.1^a^	86.9^a^	93.4^a^	98.2^b^	91.7^a^
***Anopheles gambiae***					
Total females caught	97	41	38	57	85
Average catch per night	2.7^a^	1.1^b^	1.1^b^	1.6^bc^	2.4^ac^
% deterrence	-	57.7	60.8	41.2	12.4
Total females exiting	79	34	33	56	75
% exiting	81.4^a^	82.9^a^	86.8^a^	98.3^b^	88.2^a^
***Anopheles arabiensis***					
Total females caught	483	369	533	573	424
Average catch per night	20.1^a^	15.4^a^	22.2^a^	23.9^a^	17.7^a^
% deterrence	-	23.6	0	0	12.2
Total females exiting	392	319	469	450	352
% exiting	81.2^ac^	86.4^ab^	88.0^b^	78.5^c^	83.0^ac^

Numbers in the same row sharing a letter superscript do not differ significantly (P >0.05)

Exiting rates of *An. gambiae* and *An. funestus* from huts were high with untreated nets (81 and 90 %, respectively). A significant insecticide-induced exophily occurred for both species only with the CTN washed to the cut-off point (P = 0.02 for *An. gambiae* and P = 0.003 for *An. funestus*). The majority of *An. arabiensis* (81 %) exited the control huts during the night, and no insecticide-induced exophily was apparent.

#### Mortality and overall killing effect

**Fig. 2:**
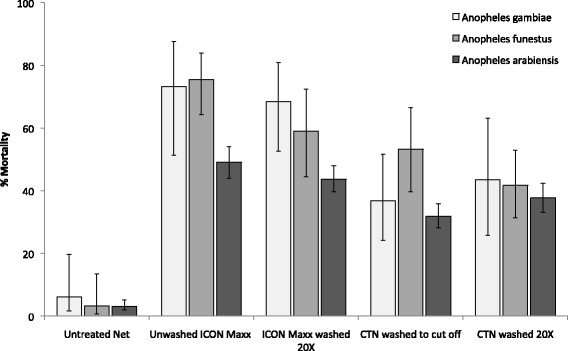
Percentage mortality of *Anopheles gambiae*, *Anopheles funestus* and *Anopheles arabiensis* in experimental hut trials of ICON Maxx treated nets and lambda-cyhalothrin CTN

Percentage mortality by treatment is shown in Fig. 2 and mortality corrected for control and overall killing effect is shown in Table 2. With *An. gambiae*, mortality with ICON Maxx treated nets was not significantly less at 20 washes (66 %) than at zero washes (71 %) (P = 0.95) and was twice as high as the mortality observed with CTN washed to cut-off point (33 %) (P = 0.001). Unwashed ICON Maxx treated nets induced 75 % mortality of *An. funestus*. The mortality of *An. funestus* was not significantly higher with ICON Maxx washed 20 times compared with the conventionally treated nets washed to the cut-off point (58 and 52 %, respectively; P = 0.058). During the Moshi trial, the mortality of *An. arabiensis* with ICON Maxx treated nets washed zero times (47 %) and 20 times (42 %) were significantly higher than the mortality observed with conventionally treated nets washed four and 20 times (30 %, 36 %). No significant difference in mortality was observed between unwashed and washed nets of either treatment. With the CTN 20 times washed, considerable mortality was still observed across all three species, ranging between 36 and 40 %.

**Table 2:** Mortality and blood-feeding outcomes of anopheline mosquitoes collected in the ICON Maxx experimental hut trials in Muheza and Moshi, Tanzania in 2008

	Untreated net	ICON Maxx	ICON Maxx	CTN	CTN
Number of washes	0	0	20	Cut off	20
***Anopheles funestus***					
Total dead	8	92	45	89	91
% mortality corrected for control	0^a^	74.6^b^	57.7^bc^	51.6^c^	39.6^d^
% overall killing effect	0^a^	37.8^bc^	16.7^b^	34.5^c^	37.4^bc^
Total blood-fed	81	23	20	41	88
% blood-feeding inhibition	0^a^	48.3^b^	27.9^b^	32.7^b^	0^a^
% personal protection	0^ab^	71.6^c^	75.3^c^	49.2^ac^	0^b^
***Anopheles gambiae***					
Total dead	6	30	26	21	37
% mortality corrected for control	0^a^	71.4^b^	66.3^b^	32.7^c^	39.8^c^
% overall killing effect	0^a^	30.4^b^	25.3^b^	19^b^	39.2^b^
Total blood fed	48	3	10	25	34
% blood-feeding inhibition	0^a^	85.2^b^	46.8^a^	11.3^a^	19.2^a^
% personal protection	0^a^	93.8^b^	79.2^bc^	47.9^ac^	29.2^a^
***Anopheles arabiensis***					
Total dead	15	181	233	183	160
% mortality corrected for control	0^a^	47.4^b^	41.9^b^	29.8^c^	35.7^c^
% overall killing effect	0^a^	34.4^b^	45.1^b^	34.8^b^	30^b^
Total blood fed	131	40	44	52	54
% blood-feeding inhibition	0^a^	60^bc^	69.6^b^	66.5^bc^	53^c^
% personal protection	0^a^	69.5^b^	66.4^b^	60.3^b^	58.8^b^

Numbers in the same row sharing a letter superscript do not differ significantly (P >0.05)

As a significant deterrence effect was observed with most treatments against *An. gambiae* and *An. funestus*, the overall killing effect was usually less than the percentage mortality of mosquitoes collected from the huts except with the CTN washed 20 times, which showed no deterrence effect. The overall killing effect was similar across most treatments because there was a trade-off between high mortality and high deterrence with the ICON Maxx treatments and low mortality and low deterrence with the CTN treatments. As no significant deterrence effect was observed against *An. arabiensis*, the overall killing effect and percentage mortality were quite similar to each other. The majority of dead mosquitoes were collected from window and verandah traps rather than the room.

#### Blood feeding inhibition (BFI) and personal protection

**Fig. 3:**
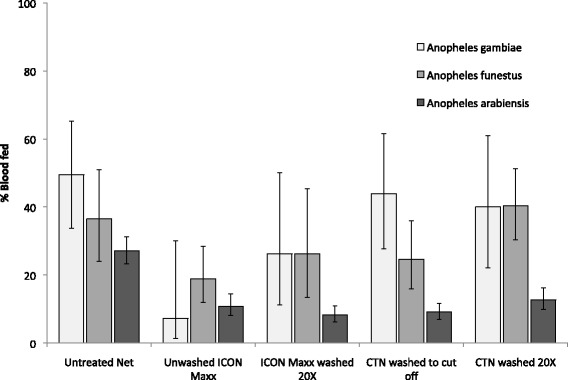
Percentage blood feeding of *Anopheles gambiae*, *Anopheles funestus* and *Anopheles arabiensis* in experimental hut trials of ICON Maxx treated nets and lambda-cyhalothrin CTN

Percentage blood feeding by treatment is shown in Fig. 3 and blood-feeding inhibition and personal protection is shown in Table 2. In the Muheza trial, significant blood-feeding inhibition was observed in both species with ICON Maxx treated nets unwashed or 20 times washed but BFI was generally less in *An. funestus* (48 and 28 %, respectively) than in *An. gambiae* (85 % and 47 % respectively). Blood-feeding inhibition of the ICON Maxx treated nets 20 times washed was not significantly different to that in the CTN washed to cut-off against either *An. funestus* (28 *vs* 33 %, P = 0.247) or *An. gambiae* (47 *vs* 11 %, P = 0.173). In the Moshi trial, all insecticide treatments provided significant blood-feeding inhibition (ranging from 53 to 70 %). Blood-feeding inhibition for ICON Maxx treated nets 20 times washed was similar to that of the conventionally treated nets washed to the cut-off point (70 and 67 %, respectively).

The personal protective effect against the biting *An. gambiae* was 79 % with the ICON Maxx treated nets 20 times washed and 48 % with the CTN washed to cut-off (P = 0.059). Against *An. funestus*, these values were 75 and 49 %, respectively (P = 0.114), and against *An. arabiensis* they were 66 and 60 %, respectively (P = 0.395).

### Chemical analysis

The chemical analysis (Table 3) showed that mean (±95 % CI) lambda-cyhalothrin content of ICON Maxx and CTN samples was 59.7 ± 29 mg/m^2^ and 13.2 ± 6.1 mg/m^2^, respectively. Both means were close to the target application rates of 55 mg/m^2^ and 15 mg/m^2^, respectively. Twenty washes removed 48.5 % of lambda-cyhalothrin from the ICON Maxx netting and 98.5 % from the CTN. The lambda-cyhalothrin remaining on the CTN washed to cut-off was 3.8 % (0.5 mg/m^2^) and on ICON Maxx washed to cut-off (27 washes) it was 10.5 % (6.3 mg/m^2^).

**Table 3:** Chemical analysis of lambda-cyhalothrin on the ICON Maxx and CTN in the experimental hut trial in Muheza, Tanzania in 2008

Number of washes	Concentration of alpha-cypermethrin (mg/m^2^)	
	ICON Maxx	CTN
0	59.7 ± 29.1	13.2 ± 6.1
4^a^	-	0.5 ± 0.1
20	29.0 ± 18.3	0.2 ± 0.1
27^b^	6.3 ± 3.3	-

^a^cut-off wash number for CTN

^b^cut-off wash number for ICON Maxx

### Supporting bioassay tests on ICON Maxx nets and CTNs used in the trials

#### Cone bioassay tests

ICON Maxx and lambda-cyhalothrin CTN nets were tested by cone bioassay using *An. gambiae* Kisumu on five sections of the net (n = 50) before washing, after washing 20 times (before the trial) and after the hut trial. Before washing, mortality was 100 % for both treatments. After washing, the ICON Maxx and CTN induced 88 and 50 % mortality, respectively, and at the end of the trial they induced 92 and 12 %, respectively.

#### Tunnel tests

**Fig. 4:**
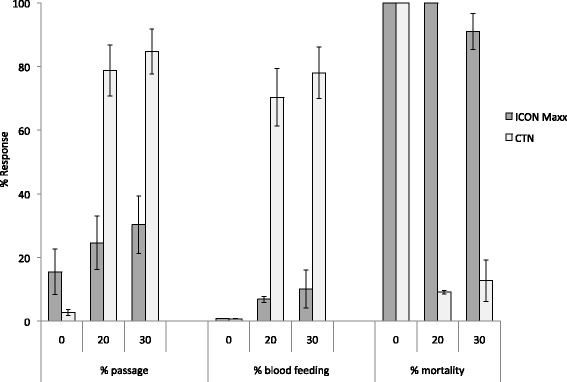
Tunnel test results with ICON Maxx and lambda-cyhalothrin CTN before and after washing against *Anopheles arabiensis* Doldotha pyrethroid susceptible strain

Tunnel tests using *An. arabiensis* Doldotha (pyrethroid susceptible) strain on ICON Maxx and CTN netting washed zero and 20 times are shown in Fig. 4. The proportion penetrating the unwashed ICON Maxx and CTN netting was less than 20 %, the proportion killed was 100 % and the proportion blood-fed was less than 2 %. With 20 washes, the proportions penetrating the ICON Maxx and CTN were 25 and 79 %, the proportions blood feeding were 10 and 78 %, and the proportions killed were 100 and 9 %, respectively. In all three criteria ICON Maxx was significantly superior to the CTN (p < 0.01).

## Discussion

The Phase II experimental hut trials performed in Tanzania on anopheline populations susceptible to lambda-cyhalothrin (*An. funestus*, *An. arabiensis* and *An. gambiae*) demonstrated that ICON Maxx induced significantly higher mortality and similar rates of blood-feeding inhibition compared to a conventional lambda-cyhalothrin treated net washed to cut-off, and therefore fulfilled the WHOPES criterion of a long-lasting insecticidal treatment. In a further WHOPES-supervised Phase II trial in Burkina Faso against a population of *An. gambiae* that was pyrethroid resistant the mortality induced by unwashed Icon Maxx against free-flying mosquitoes was less than 30 % compared to the 71 % mortality generated against the population in Tanzania, which were susceptible [12]. Despite the high-level of pyrethroid resistance (due to *knock*-*down resistance* frequency of 0.7-0.9 and probably metabolic mechanisms too) in Burkina Faso and the low mortality recorded in the trial, Icon Maxx was shown to be superior to the CTN washed to cut-off in the huts [12]. Given the efficacy and resistance to washing of nets treated with ICON Maxx in both studies, WHOPES recommended that an interim recommendation be granted to ICON Maxx as a long-lasting treatment [12]. The one caveat was the nets sampled in Burkina Faso did show an unexpectedly high variation both between and within nets and therefore WHOPES concluded that given the heterogeneity in lambda-cyhalothrin concentration on the surfaces of the nets, ICON Maxx cannot be recognised as equivalent to a WHOPES-recommended, factory-produced LLIN where greater attention can be paid to quality assurance during production. Because only a limited number of nets could be analysed chemically in the Tanzania trial, it was not possible to assess variation in application rate to the same degree as in the Burkina Faso trial. Overall, the lambda-cyhalothrin retention index after 20 washes in the Tanzania and Burkina Faso trials was 51.5 and 28.2 %, respectively, and both of these were significantly superior to the CTN retention index. Crucially, biological performance against free-flying anophelines did not significantly deteriorate after 20 washes in either trial and therefore any heterogeneity in concentration across the surface of the net does not translate to a loss of biological efficacy if mosquitoes are sampling a range of insecticide concentrations across the surface as they attempt to gain access to the host. A third experimental hut trial was conducted with ICON Maxx in Côte d'Ivoire in which over 60 % of free-flying *An. gambiae* were killed but as the resistance status was undetermined this result is difficult to put into context [22].

The WHOPES guidelines for testing of LN were revised in 2013 to include as a positive control a WHOPES-recommended LN with similar specifications to the candidate LN in the type of insecticide, treatment technique, netting material and wash number (0 and 20 times) [23]. LN manufacturers are not necessarily keen to have their established LN product compared against another LN and, by necessity, the reference LN often needs to be obtained from the free market. Some recent WHOPES trials have re-instated the CTN washed to cut-off, in addition to the reference LN washed 20 times, as a second comparison arm to check that the equivalence/superiority of the reference LN is being maintained through quality assured production. The present trial was undertaken before the revised guidelines were introduced. In view of the quality assurance issues, it is important to retain the CTN in WHOPES Phase II hut trials as one of the positive control arms.

The laboratory biological and chemical assays confirmed that the ICON Maxx insecticide binding process imparts strong wash-retention characteristics. The Phase II washing regime stripped 96 % of the lambda-cyholothrin from the conventionally treated net within just a few washes as demonstrated by the surface content falling from 13.2 to 0.5 mg/m^2^ at cut-off and to 98 % reduction after 20 washes. And yet in hut trials the CTN was still killing up to 40 % of all three species of *Anopheles* after 20 washes. A similar finding was observed in Phase II experimental hut trials of Interceptor LN, with the alpha-cypermethrin CTN washed 20 times killing between 40 % and 50 % of anophelines in the hut trials [24]. The plausible explanation is that alphacyano-pyrethroids, such as lambda-cyhalothrin and alpha-cypermethrin, have strong binding affinity to polyester filaments so that even after multiple washes a thin layer of pyrethroid of less than 1 mg/m^2^, barely detectable by HPLC, must remain bound to the fibres and be sufficiently bio-available to induce mortality in free-flying mosquitoes in experimental huts. This explanation is supported by the cone test results on CTN which showed a 60 % decrease in mortality over the first four washes and then little or no further decrease in mortality in tests over the next 20 washes.

Lower rates of mortality were recorded in the huts with *An. arabiensis* than with *An. gambiae* and *An. funestus*. Differential mortality between these species has been observed before with other types of pyrethroid in other trials of ITNs [25]. *Anopheles arabiensis* is less anthropophilic than *An. gambiae* and *An. funestus* and the favoured hypothesis is that *An. arabiensis* is likely to be less persistent at the surface of the net and more likely to be repelled by the pyrethroid. There was no evidence that *An. arabiensis* is more resistant to lambda-cyhalothrin than *An. gambiae* or *An. funestus* or shows differential response to ITN bioassay, as all three species showed greater than 95 % mortality in 3-min cone tests [25]. The lower mortality of *An. arabiensis* has been proposed as a possible explanation for the species shift in favour of *An. arabiensis* over *An. gambiae*, which has coincided with the universal coverage campaigns of LLINs in Tanzania in recent years [26].

The demonstration of retention of efficacy and wash fastness with ICON Maxx raises the prospect of long-lasting pyrethroid treatment of textile materials other than mosquito nets, such as curtains, canvas tents or blankets either in or outside the factory. There is great diversity in the fabrics and materials used for making mosquito nets; insecticide-treated blankets, tents and curtains have also shown protection against malaria in trial settings [27–29]. The, as yet, unexplored question is whether this formulation makes other types of material long lasting. The efficacy and wash resistance of ICON Maxx therefore needs to be confirmed on materials made from other types of polymer such as cotton, nylon and polyethylene before it can have the widest possible application or impact against malaria.

In Phase III trials, recently completed, ICON Maxx demonstrated efficacy criteria expected of long-lasting net after 30–36 months of household use, whereas the CTN fell short of the efficacy criteria within just 12 months of use [30]. WHOPES distinguishes between long-lasting insecticide treatments that are carried out in the community and LLINs that are produced in the factory and expected to meet higher standards of quality control and homogeneity of application [12]. The Phase III trial of ICON Maxx, recently completed by NIMR/LSHTM in Muheza, Tanzania, was the first demonstration of a long-lasting treatment, as opposed to a long-lasting factory-treated net, providing efficacy and wash fastness over the three-year expected lifetime of the net [30]. The outcome of the present Phase II experimental hut trial, with no significant loss of efficacy of ICON Maxx between zero and 20 washes, successfully predicted the outcome of the three-year household trial.

## Conclusion

Consequent to this Phase II experimental hut trial, ICON Maxx obtained interim approval from WHO and has since achieved full recommendation after Phase III household trials. It is the first long-lasting treatment kit to obtain full WHOPES recommendation.
